# Children as an afterthought during COVID-19: defining a child-inclusive ethical framework for pandemic policymaking

**DOI:** 10.1186/s12910-022-00866-w

**Published:** 2022-12-05

**Authors:** Sydney Campbell, Franco A. Carnevale

**Affiliations:** 1 VOICE Childhood Ethics Research Team, McGill University, Montreal, QC Canada; 2 Institute of Health Policy, Management and Evaluation, University of Toronto, 155 College Street, 4th Floor, Toronto, ON M573M6 Canada; 3 Joint Centre for Bioethics, University of Toronto, Toronto, Canada; 4 Ingram School of Nursing, McGill University, Montreal, QC Canada

**Keywords:** COVID-19, Child health, Pandemic policymaking, Public health, Ethical framework

## Abstract

**Background:**

Following the SARS pandemic, jurisdictions around the world began developing ethical resource allocation frameworks for future pandemics—one such framework was developed by Thompson and colleagues. While this framework offers a solid backbone upon which decision-makers can rest assured that their work is driven by rigorous ethical processes and principles, it fails to take into account the nuanced experiences and interests of children and youth (i.e., young people) in a pandemic context. The current COVID-19 pandemic offers an opportunity to re-examine this framework from young people’s perspectives, informed by advances in childhood ethics and children’s rights.

**Main body:**

In this paper, we revisit the Thompson et al. framework and propose adaptations to the ethical processes and values outlined therein. This work is informed by expertise in clinical ethics and literature related to impacts of COVID-19 and other pandemics on the health and well-being of children around the world, though with particular attention to Canada. During the processes of drafting this work, stakeholders were consulted—aligned with the approach used by Thompson and colleagues—to validate the interpretations provided. We also propose a new principle, namely practicability, to indicate the complex balance between what is possible and what is convenient that is required in ethically sound decisions in the context of services affecting young people. We outline and discuss the strengths and limitations of our work and indicate next steps for scholars in the areas of childhood studies and child health.

**Conclusion:**

Efforts to ensure frameworks are truly child-inclusive should be the status-quo, so pandemic impacts and policy implications can be considered in advance of emergency preparedness contexts.

## Background

The impacts of the COVID-19 pandemic have been devastating for virtually everyone around the globe. At the time of writing, in Canada alone there have been over 1,500,000 cases and over 27,000 deaths [1]. While individuals aged 70 or older have faced the most severe transmission-related risks, comprising approximately 85 per cent of COVID-19 related deaths in Canada [1], emerging research [2–6] has continued to show that children and youth (henceforth: young people) are facing significant and complex harms. Economic and physiological risks have been identified and positioned as scientific, public health, and research priorities, but examining COVID-19 impacts on young people has not been afforded sufficient national and international attention and urgency. Moreover, ethical concerns regarding young people’s COVID-19 related impacts and optimal approaches for reconciling these concerns have been inadequately examined [3]. Therefore, in this paper, we argue that decision-makers and public health officials have a moral responsibility to better address young people-related ethical concerns regarding pandemic planning efforts and discussions related to COVID-19 and any future pandemics.

In the highly-cited article by Thompson and colleagues from 2006 [7], scholars posit an ethical framework that was developed to “guide pandemic planning in hospitals” (p. 2). This was the first in-depth analysis of “the ethics *in* pandemic planning”, whereby general ethical processes and principles were identified and integrated with one another to form a comprehensive framework applicable to care and policy for pandemic planning [7]. However, as other scholars, particularly Nicholas et al. [8], have highlighted, Thompson et al.'s framework would have to be reinterpreted to consider young people’s perspectives and interests in order to understand, define, and draw lessons from the framework’s principles. And yet, a reinterpretation of the Thompson et al. framework has not been undertaken and neither has the development of an ethical framework that holistically addresses concerns relating to young people as members of a population in the context of a pandemic, that could draw on advances in childhood studies and childhood ethics. Despite acknowledgement and calls from scholars [8], these advances have been missing, leading to a large gap within pandemic planning literature and practice.

In this paper, we propose an adaptation to the pre-existing Thompson et al. [7] ethical framework that is attentive to the experiences of young people to help ensure that they are adequately recognized and that their particular concerns are incorporated within pandemic policies and planning. What we propose also indicates the various ways that decision-makers and public health officials can take action in an ethically sound manner to address pandemic concerns specific to young people. The purpose is not to override or challenge the foundational work that has already been completed by Thompson et al., but to layer a young person focus onto the framework to demonstrate how a collective, population-based approach can and should include young people and their concerns.

We begin with an overview of the Thompson et al. framework followed by a description of a childhood ethics framework and relevant children’s rights literature—both of which have informed our analysis. We then revisit the Thompson et al. framework to both re-envision existing ethical principles and processes and articulate a new principle that aligns with a child-inclusive focus. We highlight underlying takeaways from these proposed modifications and provide two examples of how the framework can be applied in cases adapted from our practice and research. Finally, we highlight next steps and provide concluding remarks.

## Main text

### Learning from the SARS pandemic

Thompson and colleagues developed an ethical framework for future pandemic influenza crises, drawing from Toronto’s experiences during SARS, and sought to “encourage reflection on important values, discussion and review of ethical concerns arising from a public health crisis” (7, pg. 4); during the COVID-19 pandemic, this framework has been used and cited in academic publications [9, 10] and institutional policy tools/guidance [11–13] around the world. The framework involves five ethical processes and ten ethical values to ensure a robust plan for policy response within a pandemic.

The **ethical processes** were informed by the “accountability for reasonableness” model, as defined by Daniels and Sabin [7, 14, 15]. According to this model, there are five key ethical processes for health care priority setting. Per Thompson et al. [7], these processes include^1^:

1. Accountability
2. Inclusiveness
3. Openness and Transparency
4. Reasonableness
5. Responsiveness

The **ethical values** (or **principles**) used within the framework are meant to guide pandemic planning processes and decisions, though the authors acknowledge that ethical concerns often arise from a tension between conflicting values and the challenge in identifying an ethical value that ought to take priority in a certain situation [7]. The values were informed by predecessors who attempted to highlight the ethical values that should be granted priority during the SARS epidemic in 2003 [16], which were further described and expanded upon in this framework [7]. The final list of values articulated in this framework (see footnote 1) were:

1. Duty to Provide Care
2. Equity
3. Individual Liberty
4. Privacy
5. Proportionality
6. Protection of the Public from Harm
7. Reciprocity
8. Solidarity
9. Stewardship
10. Trust

The framework was reviewed by key stakeholder groups, namely senior administrators at Sunnybrook and Women’s College. Thompson et al. indicated that future ethical framework analyses ought to engage with patient and family representatives for input as this could not be completed in the scope of their project [7]. The importance of engaging stakeholders in framework development was a key takeaway from Thompson et al.'s project, as it indicated the ways in which an ethical framework can be made more relevant and have a greater utility, and the ways in which engagement in framework development raised an opportunity for broader ethical dialogue about pandemic planning [7].

We chose to use Thompson et al.’s work as a starting point based on the immense uptake of this framework, especially in institutional policymaking. Moreover, many of the processes and values identified in this framework align with our approach to and vision for the well-being of young people and, as subsequently described, the theoretical understanding of childhood that our work employs. Despite the strengths of this framework for senior administrators in hospitals and public health agencies tasked with pandemic resource allocation, there are limitations in how Thompson et al.’s framework addresses the interests of young people. Particularly, the applications of the ethical processes and values could take on new forms or new meanings when applied in a pediatric context or when interpreted with a child-inclusive lens [8]—as discussed below. In addition, there are concerns that may be unique to young people and necessary to consider in a comprehensive pandemic ethics framework that applies to all members of a population, which may influence how specific values should be interpreted in specific situations involving young people. In what follows, we outline the ways that we have re-interpreted the values with two child-inclusive lenses and added to the framework to promote recognition of the interests of young people in a pandemic context—drawing from the current, albeit limited, literature.

### Interpretive lenses for analysis

#### Childhood ethics

To help orient pandemic standards and decision-making in a manner that is optimally inclusive of young people’s interests and concerns, we draw on a childhood ethics framework and the children’s rights literature. Childhood ethics calls for shifts that build on advances in the field of childhood studies [17, 18]. Specifically, these shifts include changing the ways we understand: childhood, social/human sciences research, ethics research and practice, and interdisciplinary collaboration [17, 19]. Young people are viewed as agents with rights, capabilities, meaningful aspirations, moral concerns and voices to contribute to discussions and decisions that affect them [17], contesting dominant “developmental” conceptions of children as “immature” or “incapable”. Here, our definition of agency aligns with that outlined by Montreuil and Carnevale [20], whereby children’s agency refers to “children’s capacity to act deliberately, speak for oneself, and actively reflect on their social worlds, shaping their lives and the lives of others” (p. 510). Children use many different tools to realize their agency, such as bodily expression, and they can impact their social circles as a result of their agency. Within the childhood ethics framework, childhood itself is seen as a social construct, whereby the meaning of childhood shifts across time, space, cultures, and individuals. Likewise, the concept of childhood agency is not static and it shifts over time and according to context (including in a pandemic); a concept analysis of children’s agency in a pandemic is outside the scope of this paper, but would be a useful topic to explore in the future.

At its foundation, childhood ethics relies on a hermeneutic ethical lens to orient how we understand matters affecting young people, value their voices, and interpret their concerns and experiences. The experiences of young people can be understood from various disciplinary perspectives, rooted in insights from young people themselves who help shape these reflections and analyses.

In this paper, childhood ethics is used to inform a re-interpretation of pandemic ethical values and processes, by asking questions such as: how is the agency of young people recognized (or not), are the known or anticipated interests^2^ of children being sufficiently met, in what ways might an ethical value overlook the rights and agency of children (and in what ways does/can an ethical value recognize this agency), and how are the interests of children—as a sub-population within a collective—taken seriously (and/or how can they be taken seriously).

#### Children’s rights

A children’s rights lens can further bolster approaches to pandemic planning and resource allocation. The United Nations Convention on the Rights of the Child (CRC), the most widely ratified treaty in history [21], outlines young people’s political, civil, cultural, economic, and social rights [22]—which are protected under international law [21]. These rights are categorized into three groups: (a) participation rights, (b) rights related to protection from and prevention of abuse, neglect, discrimination, exploitation, and other harms, and (c) rights related to the provision of assistance to support basic needs that young people have [23, 24]. The CRC relies on an “interests” conception of rights, whereby any action that pertains to a young person must hold the young person’s ‘best interests’ as a primary consideration [24, 25].

There is much debate around what a young person’s best interests are or how best interests ought to be defined. In this analysis, ‘best interests’ is imagined in terms of: a non-universal definition of best interests for young people, attuned to the particular social contexts young persons are situated within, and a recognition of young people’s voices and agency as crucial sources for informing the discernment of their best interests [19].

Utilization of a child rights lens is vital here because it promotes societal recognition, particularly by policymakers, parents, or other adult counterparts, that young people are agents with rights rather than objects to be maneuvered [26]. It emphasizes that children’s rights are human rights that can be demanded, rather than forms of charity or pity [27]. It also complements the ethical framework by grounding the re-envisioned ethical values in international law that almost every country has formally recognized and agreed to.

### Method

To begin our critical analysis of the Thompson et al. framework, we scanned existing and emerging pandemic literature to review what had already been written about the key ethical dimensions that are necessary to consider when responding to child-related concerns within a pandemic context. In total, we found nine published pieces and one report. We included literature from previous pandemics, such as the severe acute respiratory syndrome (SARS) pandemic and the swine flu (H1N1) pandemic, as very little had been written about the *ethical dimensions* of pandemic responses to COVID-19 impacts that young people were facing or in settings for young people (e.g., pediatric hospitals). Our critical analysis of the Thompson et al. framework, therefore, draws on these ten documents, along with empirical work, reports, and news releases that did not explicitly employ an ethical lens, but have been collected and analyzed for other projects since June 2020 as well. We also integrated reflections and modified cases from one author’s (FAC) experiences working in a clinical role, in community settings, and as an ethicist throughout the COVID-19 pandemic. Stakeholder engagement was also sought to inform the adaptations, as prioritized by Thompson et al.’s work [7]. This included feedback from: members of a youth advisory council that is associated with the research team that the authors are affiliated with; providers and professionals in a variety of clinical and administrative roles within health care institutions; academics who work in child research settings; and parent advisors. In total, 16 individuals provided feedback on key aspects of this framework. In addition, one of the authors (FAC) previously conducted focused discussions as a clinical ethics consultant with children’s services providers in a variety of contexts, including clinical settings and community organizations. During these discussions, providers highlighted problems with current pandemic measures, which corroborated approaches developed in this paper. It is important to note that pandemic responses and management were still ongoing at the time of writing.

### Analysis

Our critical analysis of Thompson et al. [7] focused on the identified concerns relating to young people within a pandemic, while framing these concerns in alignment with the childhood ethics and children’s rights lenses described above. Tables 1 and 2 further outline our proposed adaptations and refinements to the Thompson et al. framework.

**Table 1:** Ethical processes

Process	Our adaptations	Related CRC articles	Actualizing the process
Accountability	Mechanisms to monitor pandemic-related decisions must prioritize and incorporate the rights of children into policy decisions and should assess the ways these rights are being overlooked. These assessments should be explicitly led by decision-makers, private organizations, legislative bodies, and other key stakeholders, ideally through a third-party review that ensures neutrality and fairness.	Article 3: Best Interests of the Child Article 43–45: These articles explain how governments and international agencies will work to ensure children’s rights are protected.	Children’s Rights Impact Assessments (CRIAs), a systematic process in which policies and decisions are assessed to see whether and how they impact children’s rights, are one useful method for monitoring (amongst others). CRIAs are recommended by the UN Committee on the Rights of the Child. These should also take into account the needs and rights of particular groups outlined under other UN conventions (e.g., United Nations Convention on the Rights of Persons with Disabilities, United Nations Declaration on the Rights of Indigenous Persons).
Inclusiveness	Young people must be considered stakeholders by decision-makers and society in order to be included. Their voices are often shared through the work of adults, but opportunities for direct engagement with young people (in ways that align with their interests) should be developed and implemented in pandemics. Fundamentally, these voices must be solicited and genuinely listened to in these settings, as well, and intersectional elements of identity must be acknowledged and accounted for in engagement strategies. The CRC should be reviewed and consulted to see where children’s rights are overlooked, how their lives are impacted by being excluded, and where disparities exist across groups of children.	Article 3: Best Interests of the Child Article 5: Family Guidance as Children Develop Article 12: Respect for Children’s Views Article 13: Sharing Thoughts Freely	One way to ensure that young people are included as a relevant group in pandemic policy decisions is to have regional and national children and youth advocates who oversee and examine potential policies. In these settings, young people from a variety of sociocultural and economic positions should also be consulted through paid positions in advocacy offices, discussions with youth advisory councils, and community consultations. These strategies should be informed by children and youth themselves [38–42].
Openness and Transparency	Decisions must be clear and understandable for the entire population. Decisions that are publicly accessible—ones that promote ‘health policy literacy’—require the translation of decision trails into formats that allow understanding on the part of *all* young people too. With transparency must come the possibility of criticism or suggestions from young people, as with any population, and a need for public bodies to respond to these perspectives.	Article 3: Best Interests of the Child Article 17: Access to Information	All public-facing information should be in plain language to improve accessibility amongst young people and their families. In addition, to facilitate critical dialogue and ensure understanding public roundtables, think-tanks, or town halls specifically designated for young people should be created and commonplace. Ideally these would be in public venues like schools or recreation complexes.
Reasonableness	Qualitative research that engages populations of young people, and their families, should be considered reasonable evidence for policy decisions. Young people, themselves, should also be seen as reasonable and reliable. Moreover, the scope of information being examined must also be broader. For example: many of the impacts affecting children are future-focused, though immediate impacts are prioritized in pandemic decision-making. Longer-term risks must be included in long-term pandemic plans. While this focus on present concerns is understandable, one by-product is that it may lead policymakers to disregard the ways in which adapting solutions and redistributing resources now *to mitigate ****predictable**** future harm* (for the sake of children’s futures) can have both immediate and long-term benefits for all of society.	Article 3: Best Interests of the Child Article 12: Respect for Children’s Views	To ensure all evidence is taken into account, public health scientific and bioethics tables must: (a) take a systemic approach when examining pandemic-related evidence that includes various forms of research and sources of knowledge, (b) grant proportional weight to future harms, harms indirectly related to viral transmission or enforced public health measures, and (c) ensure children-specific impacts are included, where evidence is available.
Responsiveness	We need to continue to ensure that we are responsive to the needs of young people too, especially as new information emerges about the ways they are being harmed within COVID-19 that may be outside the ‘transmission’ concerns. A formal mechanism for parents, caregivers, and young people to provide feedback must be developed in a way that is attentive to the capacities of an organization or state.	Article 3: Best Interests of the Child Article 4: Making Rights Real Article 12: Respect for Children’s Views Article 13: Sharing Thoughts Freely Article 42: Everyone Must Know Children’s Rights	Implementing a formal complaint mechanism for young people and those in their social circle could be organized by government departments that have mandates for the well-being of children, by provincial Child and/or Youth Advocates (e.g., in New Brunswick [43]) or by implementing a federal Child and Youth Commissioner. This mechanism ought to be efficient and tailored to children and families needs (e.g., open after school hours). Aside from a mechanism for reporting concerns, these bodies should also develop tools that translate the concerns of young people and their caregivers into policy-oriented solutions—again, by working with young people directly, where possible.

**Table 2:** Ethical values/principles

Value/principle	Our revision	Related CRC Article(s)
Duty to provide care	Conceptualizations of ‘good’ care need to be rooted in strong conceptualizations of best interests and agency that position young people as agents with rights. In particular, care in a pandemic needs to equally consider the young person and their capacities in the ‘here-and-now’ and their ‘future’ capacities, along with the ways in which children are interrelated with those around them. Caring for young people requires that we care for those who support them too. In addition, care should extend beyond the health sphere and acknowledge that public health policies have impacts on many other sectors, especially for children, such as education, social supports, child welfare, etc. Engaging with teachers, parents or others to establish practice guidelines that work for their particular settings or spaces is one way that care can be extended during a public health emergency. Young people should also be involved in defining what types of care they need and how the tools for care provision can align with public health goals.	Article 3: Best Interests of the Child Article 6: Life Survival and Development Article 9: Keeping Families Together Article 12: Respect for Children’s Views Article 13: Sharing Thoughts Freely Article 19: Protection from Violence Article 22: Refugee Children Article 25: Review of a Child’s Placement Article 26: Social and Economic Help Article 28: Access to Education Article 30: Minority Culture, Language and Religion Article 32: Protection from Harmful Work Article 34: Protection from Sexual Abuse Article 36: Protection from Exploitation
Equity	Social determinants of health ought to play a role in pandemic decision-making. There needs to be a place for long-term impacts to be included within pandemic plans *and* a fulsome examination of the way children's lives and rights will be impacted by pandemic decisions (and how particular children's rights, such as rights to education, may be impacted more than others by decisions made). Emphasizing the fundamental right young people have to access and receive healthcare is crucial in this context. In addition, several groups of children have experienced heightened familial and individual burden and harm, as a result of the pandemic, that have been overlooked and not rectified. To note, many of these impacts have existed before the pandemic and have been heightened in the current context, and the impacts amongst these groups are not identical.	Article 2: No Discrimination Article 3: Best Interests of the Child Article 30: Minority Culture, Language and Religion
Individual Liberty	Shifting to more relational models of liberty and autonomy is necessary, as these concepts are seen as better visions of autonomy and individual liberty for young people, considering the fundamental social relations that structure their lives. Pragmatically, this may mean acknowledging times when restrictions are necessary but not sufficient for supporting a young person accessing care by recognizing the crucial role for parents, caregivers, and family in caring for young people during a pandemic. Ideally, interdisciplinary teams will be created to support young people and families with decisions, wherein the young person is still able to contribute to deliberations on matters that impact them and where tools (like art or surveys) are used to facilitate conversation and knowledge-sharing.	Article 3: Best Interests of the Child Article 5: Family Guidance as Children Develop
Privacy	In light of young people’s immersion in the digital wave, the definition of privacy must capture the need to protect youth in online forums (i.e., on Zoom, on social media, etc.), as they frequently access these spaces. Relational views of children need to be used to contribute to understandings of privacy, whereby young people “have their own ethical interests” that have impacts on how their best interests are interpreted, but also acknowledging that young people are “embedded within sociocultural networks of morally-significant relationships” that make confidentiality and privacy complex [56p. e10]. Decision-makers must recognize the capacities of young people and their rights to privacy, while simultaneously acknowledging the protective responsibilities of parents or caregivers. For instance, young people may not want parents to be informed of decisions they make regarding choosing to be vaccinated; a balanced and collaborative approach that ensures parents are informed of urgent needs of children, while children’s rights to privacy are also ensured, is necessary.	Article 3: Best Interests of the Child Article 8: Identity Article 16: Protection of Privacy
Proportionality	Young people have faced disproportionate impacts due to COVID-19 as their current and future concerns have not been carefully attended to and the pandemic precautionary measures have unintentionally caused some problematic effects for young people. To prevent or mitigate these harms, decision-makers must consider that young people face different types of harms than adults and harms that may not have an immediate impact. Children’s rights (to education, to play, to be protected from harm, etc.) ought to be protected to the greatest degree possible or restricted to the least degree possible. Finally, acknowledging that there should be a balance between upholding the interests and rights of young people while having necessary restrictions in place, whereby young people’s needs are not treated as secondary to adults, is a way to ensure proportionality for the young population.	Article 3: Best Interests of the Child
Protection of the Public from Harm	Children have tended to face post-pandemic impacts in previous pandemics, and there are risks of this with the COVID-19 pandemic too. One study from 2013 indicated that living through a pandemic had serious mental health impacts on children, and these impactst arose after the pandemic ended, such that nearly one-third of children who experienced isolation or quarantine had symptoms that met the overall threshold for post-traumatic stress disorder [64]. Adults may face different levels of harm from certain policies compared to young people and this must be weighed when considering what harms we want to protect the public from and how to adapt for a pediatric setting; here, proportionality again becomes valuable to consider. Children also have rights to protection from harm and there are instances where these have not been abided by. Shifts to acknowledge and mitigate these other harms, through policy and practice, is necessary. Marginalized individuals and communities must be centered in these decisions, to ensure they are protected from the additional harms they face.	Article 3: Best Interests of the Child Article 9: Keeping Families Together Article 19: Protection from Violence Article 20: Children without Families Article 22: Refugee Children Article 25: Review of a Child’s Placement Article 27: Food, Clothing, a Safe Home
Reciprocity	When we consider impacts outside of transmission concerns, we recognize that children of all ages have faced and will continue to face disproportionate burden for the sake of protecting public interests and the lives of their families, friends, and loved ones, and these burdens arise in different forms. It is essential for decision-makers to keep this in mind when developing additional measures of pandemic response, both during active outbreak/community transmission and following the pandemic. It is necessary to support parents/caregivers too, since children need these individuals to have their interests realized. For example, employing paid sick-leave for parents, families, and youth working in essential jobs and in low socioeconomic positions is one step to ensuring that their current needs are met and for a response driven by reciprocity. Providing preemptive strategies of how children and their family’s economic, social, political, psychological, and physical burdens will be rectified during the pandemic and beyond is essential. Young people should be seen as partners in these conversations.	Article 3: Best Interests of the Child Article 9: Keeping Families Together Article 12: Respect for Children’s Views Article 13: Sharing Thoughts Freely Article 15: Setting Up or Joining Groups Article 18: Responsibility of Parents Article 19: Protection from Violence Article 24: Health, water, food, environment Article 31: Rest, Play, Culture, Arts
Solidarity	Efforts must be taken to ensure solidarity exists between decision-makers, clinicians, parents, and children too. In addition, child refugees and families in lower socioeconomic positions faced a heavy burden due to pandemic decisions, so including their experiences in pandemic decisions, through regional and global solidarity with these experiences, is important [77]. There must also be solidarity with the losses that young people have faced as a collective, particularly infringements on their rights as children. These rights must be treated as legally binding commitments rather than a form of ‘pity’ or ‘charity’ [27]. Young people can also help to promote solidarity in their communities, nationally, and internationally.	Article 3: Best Interests of the Child Article 12: Respect for Children’s Views Article 13: Sharing Thoughts Freely
Stewardship	Stewardship requires the particular needs of young people to be carefully considered, so that appropriate resources can be reserved for these needs. This is important as reports have indicated a large decrease in number of parents/children attending hospital for complex chronic conditions and the significant (and crucial) delays in children seeking and receiving diagnoses for their symptoms/illnesses due to COVID-19 [78]. It will be the responsibility of institutions and governments to manage resources, and this needs to be integrated with pediatric care too. Those responsible for managing resources must also consider what is necessary or valuable for the future and protect those resources, such as human potential and talent On the other hand, stewardship also requires children’s institutions to potentially provide resources for adults to use within a pandemic, in order to protect society at large.	Article 3: Best Interests of the Child Article 24: Health, water, food, environment Article 26: Social and Economic Help
Trust	Developing trust with young people may require different methods. It involves establishing trust regarding public health decisions that are made and this, inherently, requires the provision of information and communications that are young-person focused and informed. Different methods for building trust may include developing effective and individualized strategies to determine who, in a particular group of young people, can speak on behalf of the group without trivializing the voices of the population. Once these individuals are identified, shared governance or co-governance models must be established, in order for ‘adult leaders’ to ensure that young contributors feel supported, heard, and able to trust the adult partners. This process needs to start early, just as it does with adults, and relies on the use of consistency in public messaging. Learning from established Youth Advisory Councils/Committees is one way to optimize these approaches. At the same time, just as adults have the right to freely hold their own beliefs, young people too should be granted the rights to their beliefs. Establishing trust means accepting the possibility that these beliefs may not align with public health interests—though efforts should continually be made to keep young people up-to-date on the most current evidence, answer their questions, and inform young people of the consequences of their decisions. Public trust must be built over time and requires engagement with behavioural science specialists to ensure pandemic responses are fundamentally activities that promote trust [79], especially with young people	Article 3: Best Interests of the Child Article 12: Respect for Children’s Views Article 13: Sharing Thoughts Freely Article 14: Freedom of Thought and Religion Article 17: Access to Information
Practicability	[Im]Practicability lies between [im]possibility (i.e., whether we can do something) and [im]practicality (whether a thing is useful to do). It is a way of understanding the feasibility of something. By determining if something is practicable, you are determining whether it is do-able, even despite how inconvenient it may be, existing resource constraints, etc. In a time where the impacts of the pandemic on young people have been largely overlooked compared to adults and compared to controlling rates of viral transmission, it may be practicable to devote funding to ensuring that young people’s interests are going to be met. However, this may not be practical (in that there are competing demands, diverse interests, an adultcentric society, and the rational need to worry about limiting transmission as a primary concern). As such, a fine balance is required to weigh all options and grant priority to solutions that prioritize minimizing the greatest degree of harms possible and maximizing the goods. Overall, practicability requires decision-makers to: 1. Consider all of the risks and benefits that needs to be balanced and the associated minimum threshold for each risk (whereby exceeding the threshold would mean a disproportionate level of risk would be experienced). 2. Take into account whether it is *possible* to prioritize a particular action or decision, and whether all pathways towards the aim have been exhausted to determine whether something is possible/impossible. 3. Decision makers would also need to take into account whether it is useful to pursue an action/decision/end and who it may be useful for.	Article 3: Best Interests of the Child

#### Ethical processes

In examining how the ethical processes described by Thompson et al. could be adapted to better acknowledge the complex interests of young people, we began by assessing how adaptations could sufficiently capture young people’s perspectives and interests. These proposed adaptations are outlined in Table 1.

In terms of *accountability*, our adaptation highlights the need for governments, public health agencies, and health care institutions to be accountable to all stakeholders (including young people and their families) in how decisions are made that relate to the pandemic (i.e., ensuring that the public health strategies are informed by sound ethical values). This requires engaging with young people in a variety of forums to ensure public health decisions are informed by their lived experiences and for their complaints to be heard in a respectful manner. Accountability in a country that has ratified the CRC—and/or has made similar commitments to promoting children’s rights—also requires an assessment of how children’s rights are being upheld within pandemic policies. A model of accountability that prioritizes and incorporates the rights of children also positions young people as capable agents with meaningful interests.

*Inclusiveness*,^3^ from a child-inclusive perspective, must address the ways that young people have been systematically dismissed from big and small policy decisions in most countries, and the nuances in this exclusion across the entire child population based on gender, race, socioeconomic position, disability, and more. As mentioned by Daniels and Sabin [14], it is necessary to have a “broad range of stakeholders—especially consumers affected by the decisions—play the role of fair-minded individuals” when making decisions in order to “give credibility to the goal of having all relevant reasons considered” (p. 63). However, by excluding young people from various sociocultural locations not only are their interests (or “reasons") not being considered, but their capacities (or abilities to be “fair-minded individuals”) are ignored. As aforementioned, the impacts of COVID-19 on young people and their rights have not been adequately addressed in pandemic policies across most countries [26, 28], especially rights to participate and be heard [29]. Previous pandemics have taught us that this can lead to adverse impacts for young people [30]. While this exclusion is understandable, given competing interests and the need to prioritize public health interests through “utilitarian-like” models (i.e., striving to protect the greatest good for the greatest number of individuals), placing the interests of young people on the “backburner” can impact societal well-being beyond the virus’ containment [31, 32]. During the COVID-19 pandemic, researchers have heard that young people have felt inadequately or insufficiently involved in the pandemic processes within their communities, as they wish to be engaged as true partners in policy-making and implementation [33, 34]. Thus, to be truly inclusive, decision makers ought to create opportunities and spaces to ask young people if they wish to be included in pandemic planning processes, acknowledge how young people have previously been excluded accounting for intersectional elements of their identities, and develop strategies to elicit this input that align with young people’s interests, cultures, and rights. These strategies must ensure that children’s voices are granted “due weight” as outlined in the CRC Article 12 [25], and promote all young people having equitable access to decision-makers.

*Openness and transparency* are essential for ensuring the public and affected stakeholders can understand how decisions are made, understand what the decisions mean, and open decisions to critical dialogue. Importantly, as described by Daniels [15] and implied by Thompson et al. [7], openness and transparency in pandemic contexts pertains to a process of being transparent about reasons (i.e., evidence, principles, values) that “all can eventually agree is relevant” [15 p. 1301]. We need to separate this from openness and transparency about one’s intentions, which could (theoretically) be personally or selfishly motivated and yet still implicitly or explicitly informed by “reasons”—no amount of honesty about unjust or unfair intentions can make those intentions ethical. Yet, even when these decisions are publicly available, they are often not accessible for *all* young people, particularly those that are very young, with different cognitive capacities, living within diverse socioeconomic contexts, or with different interests in accessing information [35]. In examining openness and transparency against a childhood ethics and children’s rights backdrop, information released for public review should include “translations” that are adapted for utilization by all young people, including those living with communication, cognitive and/or social differences that can affect the ways they understand information. This is especially important considering the research revealing that some young people have expressed confusion or uncertainty around pandemic decisions [30, 33]. Parents/caregivers have also found it challenging to navigate these discussions with these children [36]. To open decisions to critical dialogue, the forms in which the information is released need to be optimally transparent, entailing greater comprehension for young people.

Rethinking *reasonableness* with childhood ethics and children’s rights lenses involves redefining what is included or seen as relevant, robust, or reliable information that can effectively guide stakeholder agreement and decision-making; this includes thinking about young people, themselves, as reliable sources of knowledge and as experts on matters affecting them/their own lives. Moreover, inclusive processes to generate information and define what is included must be integrated in pandemic policy planning to ensure stakeholders from various sociocultural positions are included. It is also important to widen the scope of information being examined, such that impacts that span beyond the present moment can be adequately considered. Consequently, young people may have to live with the harms (or benefits) of policy decisions made during a pandemic beyond the present moment—often well into their future—and this is often a longer period than most adults. For instance, delays in pediatric care can result in lifelong health challenges for those with pre-existing underlying conditions or those who receive a new diagnosis of a critical, complex health concern [37]. Finally, the assumption that only certain people are knowledgeable and accountable risks perpetuating a perspective where young people are globally viewed as incapable or unreasonable, and where their potential for meaningful contributions is overlooked.

Finally, changes to the ways we view *responsiveness* align with how we have envisioned change for the other ethical processes—namely, by ensuring we are consistently attending to the interests of young people, in addition to those of adults, particularly as new information becomes available and opportunities arise to revisit decisions. Additionally, as vaccination-rates increase and transmission-rates are reduced, policymakers must be willing and ready to pivot to concerns that arise due to the long-term implications associated with urgent pandemic policies, such as those associated with school closures or isolation. This vision of responsiveness will necessarily require strategies to be planned and implemented that support young people to voice their concerns and to ensure action is taken by public agencies or organizations that receive feedback from young people to meet their expressed interests and mitigate their concerns.

#### Ethical values

While there are certain criteria in place for pandemic policy processes to be ethically sound, ethical values should also be used to guide the ways that processes are designed or implemented. In what follows, we propose a re-examination of the ethical values described by Thompson and colleagues [7] using childhood ethics and children’s rights lenses, and we expand on these thoughts in Table 2. In addition, at the end of this subsection, we also highlight the need for a new value to be added to this framework, namely *practicability*.

As we re-envision the *duty to provide care* ethical value with a young person-lens, it is vital to specify what “good care” is within a pandemic when related to young people, where the concepts of best interests, agency, and capacity—as advanced within childhood ethics [19] and childhood studies [44]—are central. The value of young people as full human beings is paramount, along with the inherent relationality associated with childhood that requires public health agencies and policymakers to attend to the interests of parents/caregivers too, even when the focus is on the young person. Moreover, isolating the importance of care to being situated within the medical or health spaces during a public health emergency disregards the various spheres (e.g., schools, community organizations, recreation settings, etc.) that jointly impact children’s well-being [45] and the associated rights that encourage policymakers to attend to these interests [25].

The concept of *equity* is a particularly important concept to revisit, although we recognize Thompson et al.’s [7] definition as ethically grounded and important to retain. Core additions should involve an inclusion of children’s rights within approaches to preserve equity amongst all, especially the explicit recognition of children’s rights as human rights that require fair consideration. While we examine the ways in which young people’s rights and capacities are immediately impacted by pandemic policy decisions, we also need to be mindful of longer-term impacts that may result. For instance, the sudden closure of schools in Ontario (Canada) early in the first wave of the COVID-19 pandemic had short-term impacts on children’s mental health and socialization skills, while having potential (and predictable) long-term impacts on their economic well-being [46]. Indigenous children, Black children, and children from other racialized and/or socially disadvantaged groups have been disproportionately exposed to harms before the pandemic, which the pandemic has amplified [47–49]; this includes children with disabilities or underlying health conditions, too, who have faced additional concerns in the pandemic [50], immigrant children, and refugee children [28]. For example, there were significant challenges for children from low socioeconomic positions to gain access to computers or tablets for online schooling. For many, these impacts affect not only the young person, but also their family members [51]. These young people’s rights seem to have been overlooked to a greater degree, despite existing normative provisions that affirm their protection [25]. Thus, frameworks for addressing pandemic concerns in ethically sound ways need to be attentive to these impacts and decision-makers must actively prevent and protect young people against any form of discrimination, within and beyond the pandemic.

Attending to *individual liberty* requires a more substantive adaptation when childhood ethics and children’s rights lenses are used, considering that relational models of liberty and autonomy [52, 53] are predominantly used in discourses regarding childhood. While young people can and ought to have capacities to contribute to decisions that impact their lives and weigh in on their best interests, young people are also embedded within social contexts whereby relationships are critical to how young people navigate their worlds. Moreover, there must also be a delicate balance between public interests in a pandemic scenario and the interests of young people and their proxies; opportunities to exercise capacities or to fulfill and optimize one’s own interests may need to be restricted in a pandemic scenario, where everyone face restrictions.

Adapting the definitions of *privacy* provided by Thompson et al. requires widening how privacy is imagined to include the ways in which digital health technologies can threaten the privacy rights of young people in particular [54], especially based on the digital advances over the past two decades and young people’s increased online presence [55]. For instance, children’s decisions to download and use the COVID-19 exposure tracking application on their phones should balance their rights, their parents’ responsibilities, and their best interests. In addition, at a time when vaccine passports are a key element of discussion for how we can and/or should move forward, centering these types of discussions on privacy (as it pertains to both adults and children) is necessary. Utilizing a childhood ethics frame reminds us that young people have their own ethical interests, but they are also inherently relational which makes privacy in relation to parents/caregivers very complex [56]. There is also a concern for privacy related to vaccine choice. Legal scholars and reporters have reminded decision-makers and clinicians administering vaccines to be cognizant of the legal rights and capacities of young people in certain jurisdictions to consent to vaccination without parental consent [57], even before and beyond the COVID-19 pandemic [58]. Ultimately, young people’s privacy should be protected proportional to the risk/benefit analysis of each young person’s situation and context [56].

The definition of *proportionality* developed by Thompson et al. is based on using the least restrictive measure possible when limiting liberties [7]. However, considering that young people are predominantly deemed incapable and not allotted the same degree of liberties as adults, when it comes to a proportional balance between individual freedom and population-driven restrictions there is a pre-existing imbalance that young people face. Evidence indicates that young people have already faced disproportionate burdens within the pandemic (especially long-term harms and undermined rights) with little rectification or mitigation [3, 59]. As such, proportionality with childhood ethics and children’s rights lenses requires that we treat the liberties and rights of children with the same respect as we do with adults’ rights. We must take steps, when possible, to only restrict these rights and interests to the degree that is proportionately necessary to ensure a fair balancing of opportunities and restrictions across all groups within a population. Further, proportionality appears as a different type of value—one that trickles into and informs perceptions related to the other values—and this needs to be considered for a framework to operate effectively.

The *protection of the public from harm*, posited as a value by Thompson et al. [7], requires an examination of how we define “public” (specifically so it explicitly includes all young people and not just individuals most at risk of viral transmission, illness, or death) and an analysis of what we mean by “harm”. The latter is important because some have argued that young people have faced less risks or harms during the COVID-19 pandemic as they have not been infected with SARS-CoV-2 as frequently as adults nor have they experienced severe illness as frequently. Yet, emerging evidence from the current pandemic [2, 3, 60] and evidence from previous pandemics [61–64] has indicated that young people do face significantly harmful pandemic impacts, especially regarding their mental health and risks of long-COVID [65]. Children have rights to be protected from pandemic harms to an optimal extent. But, upholding theses rights has not seemed to be a chief concern during COVID-19, especially in resource-limited settings [28]. As such, a child-inclusive version of this ethical value needs to be focused not just on transmission-related risks and harms, though these are evidentially critical to contain and mitigate, but also on the other immediate and long-term harms that young people in particular may face.

*Reciprocity* from a child-inclusive perspective, similar to the previous value, also requires a reassessment of what is meant by disproportionate harm. If disproportionate harm is defined solely in terms of how a sub-population or group has done with respect to viral transmission outcomes, then young people may not be considered disproportionately harmed, especially within the first waves (with exceptions made for children with underlying conditions that make exposure more high risk and the increasing rate of child infection due to the Delta variant). However, in the bigger picture young people have faced disproportionate harms due to irreversible impacts caused by the pandemic and pandemic-related polices [4] and these harms arise in different forms depending on the particular group or individual being considered. As such, decision-makers should acknowledge the relational aspects of childhood, account for the ways in which young people have had to demonstrate resilience for the sake of their families, friends, and communities while bearing significant harms, and support them for being disproportionately burdened. Decision-makers should also develop pre-emptive or anticipatory strategies, rather than reactive strategies [66], for how young people’s burdens will be redressed during and beyond the pandemic.

*Solidarity* with childhood ethics and children’s rights lenses requires not only open communication between countries, governments, and institutions, but also open and accessible communication with the public in various formats to ensure *all* members of the public (including parents/caregivers, educators, providers, and young people) are informed and engaged. Several reports have indicated that this open dialogue and information was missing for young people [33], yet it is crucial for their well-being within the pandemic [67, 68]. In addition, there needs to be solidarity for the varying levels of impact that certain young populations can experience and the way these groups’ rights, as young people, have been markedly affected [69]. Children and youth should be included as consultants to help develop strategies that ensure solidarity and open dialogue.

*Stewardship* in a child-inclusive approach requires “integrated, rather than siloed, resource allocation” [70] that is managed by the state and public health units. This means ensuring continued resource investment for the care of young people (especially those with underlying health conditions or health concerns), while also supporting child health care institutions to share resources with communities or institutions that serve adults, where there may be higher hospitalization rates [71]. In a pediatric care setting, this may manifest in a way that involves exploring how ventilators are shared amongst infants and children, and how some may be allocated for adults—considering factors including who is most “at risk” of death without a ventilator, who would have the best quality of life, etc. Discussions of this sort occurred, for example, at Montreal Children’s Hospital and the Hospital for Sick Children in Toronto. Approving and delivering COVID-19 vaccinations for adults much earlier than vaccines for children is another situation that requires careful resource balancing (personnel, research investments, funding, etc.) and stewardship. Stewardship also means protecting resources that have more value in the long-term (e.g., access to high-quality education or economic investments for children), and considering ways to protect young people against harms that will have more delayed  effects. A truly population-based model, like the one we are trying to advance, encourages shared resources, whereby the process of sharing resources is stewarded by the state and integrated across areas of care, facilities, and sectors.

*Trust* with young people may involve an explicit recognition of their rights, so they feel that those in positions of authority see them as persons with interests that require protection. For young people, who often face ageism as a function of societal priorities, approaches informed by “cultural safety” advances [72] are required to acknowledge and create strategies to address power imbalances between adults/providers and children. Different methods, aside from press briefings, news releases, and reports, may be necessary to support young people as stakeholders and secure their “buy-in” for evidence-informed control measures; these methods need to also continue beyond the pandemic so trust can be built over time. Building trust also involves conscious and consistent efforts to set aside prejudgements of another individual or community’s decision/actions, in order to have meaningful conversations; this is especially important in clinical encounters, where the trust that is necessary for a fruitful relationship is not developed in the same way that occurs for “ordinary” relationships [73]. Child and Youth Advisory Councils/Committees, especially those with decades of experience, can provide insight into how to enhance strategies to build trust with young people in relation to pandemic policy development. Trust strongly relates to inoculation efforts too, whereby “establishing public trust is now central” [74] to conversations related to childhood vaccines. Considering that there is often an absence of leaders and spokespeople that are trusted by young people, efforts to develop public trust are essential. Trust, in this case, can be multifaceted, particularly when parents/caregivers are involved and a level of acceptance amongst more than one party must be achieved [74].

*Practicability* is a new ethical value that we have added based on our experiences examining, in both literature and through clinical ethics consultations, the impacts of the pandemic on young people. Practicability (or impracticability) is a way of understanding the feasibility of something based on whether that thing is possible (i.e., achievable) and/or practical (i.e., convenient). When an action is practicable, that means it is do-able, even if it is challenging to actually achieve. This value is important for operationalizing proportionality and, in a way, for operationalizing the optimization of all other values within the framework too. For example, some decision-makers may favor maximizing restrictions—to ensure prevention of viral transmission to the greatest degree possible. However, such a view can be ethically problematic because it tacitly entails that immediate biological harms are the only ones that merit consideration, thereby subordinating and disregarding all other harms (e.g., adverse social impacts or long-term biological harms).

In a truly proportional pandemic ethics approach, all pandemic-related impacts warrant attention and solutions are preferred when all harms and/or benefits—that have been deemed meaningful *by those affected*—are considered within the plan for recovery. Moreover, the pandemic measures or restrictions that are favoured, in a proportional approach, are those that minimize all harms—whereby we ensure that minimum risk thresholds are protected for all (or as many as possible) potential harms/risks, no matter the scenario. What could this minimum threshold look like for the other crucial risks borne by children in a pandemic? Well first, this would need to be corroborated with the communities in question, but from early evidence [3, 75] one could say that this may involve: no inconsolable sustained psychological/emotional distress is borne by any child because of a physical distancing measure; no physical separation between a child and their parents/caregivers in order to ensure the child’s right to parental authority representation and protect the determination of their best interests and their continual consent to care; or no academic/learning/language development setback is borne by a child that would be irremediable or would be borne inequitably by already disadvantaged young people. These thresholds also depend on there being means (even if they are inconvenient) that could help protect children from these harms in a manner where viral transmission risks can also be reasonably minimized.

To ensure a “just” and proportionate balancing of these risks (and benefits), all measures need to be considered to optimize this balance among those who are impacted—ensuring no minimum risk threshold is breached, even in circumstances where resource allocation in a clinical context or elsewhere is operating at a minimum risk threshold *prior to* a pandemic. In considering “all measures” this includes consideration of measures that may be considered cumbersome or costly. Some measures may be “impossible” (e.g., immunizing an entire population immediately with a 100% effective vaccine upon identification of a new virus of concern). Some measures may be “impractical” (e.g., closing all food and drug stores for a minimum of two weeks so communities can be shutdown more completely). As such, practicability truly falls between what is possible (what can be done, regardless of effort or costs) and what is practical (what can be done within reasonable effort/cost efficiency considerations). Practicability implies an action is “do-able” but may entail some exceptional/extraordinary inconveniences.

For example, in some contexts, parents were denied access to their disabled children in long-term care settings for several weeks or months [76]. Although this measure could help reduce viral transmission, it disproportionately superseded other vital child interests regarding access to parenting and parental representation in decisions that affect these children. Several children’s hospitals demonstrated that an essential level parental contact could be maintained safely through policies that restricted access to one parent at a time—whose movements within the hospital were restricted—along with staff or volunteer support for parents on the use of protective equipment. In this case, it was practicable to safely balance viral control with parental access.

### Discussion

This paper provides new insight into ways that a child-inclusive lens can be incorporated within predominant ethical frameworks, which can help expand current decision-making tools and perspectives. There are also several overarching reflections that we have drawn from the process of constructing this adapted framework. Our team’s work throughout the COVID-19 pandemic, and in constructing this framework in particular, has highlighted a temporal element that is fundamental to the ways decisions are made in a pandemic and which impacts become priorities for decision-makers. With good reasons and intentions, the immediate physiological and economic impacts have dominated policy agendas since March 2020 and continues to do so as COVID-19 transmission declines as a perceived societal threat. However, the impacts most prominently affecting the majority of young people (though there are some exceptions) are largely future-focused—including inadequate education, delays in medical procedures, mental health impacts, socialization disruptions, economic impacts from job opportunity losses, etc. Some of these impacts will become biologically engrained in the lives of young people, with some effects surfacing many decades from now [80]. While it is always important to focus ample attention on driving down viral spread and limiting infection as much as possible in a pandemic, the prevailing and consuming prioritization of immediate impacts will perpetuate long-term impacts for young people. Importantly, the tendency to be focused on immediate impacts is not a pandemic-specific phenomenon—this is a systemic concern that young people face and our recognition of this temporal-mediated inequity during COVID-19 points to the necessity of future research and action to address this concern. Therefore, in this adapted framework, most of the changes have emphasized a need to focus on the future more thoroughly in current decisions and to respond pre-emptively, as to anticipate threats young people will face, in order to prevent these impacts rather than respond to the concerns when they are inevitable.

Another overarching takeaway from this framework has been the crucial nature of engaging with young people as real contributors in pandemic policy conversations, just as we would expect decision-makers to do with experts in other areas. When we refrain from engaging with young people, we risk developing solutions that do not attend to the needs and concerns faced by young people. Engaging with young people entails that there are spaces to share perspectives that are open and accessible to young people, and that decision-makers also make themselves available to listen with intention to young people’s voices and experiences. To be clear: young people are experts in being able to explain what they are experiencing and the impacts they are facing. So it is reasonable to include these perspectives in a collective model for pandemic policy responses. Decision-makers must recognize that interpretations of what is in a child’s best interests may differ depending on who is asked (such as parents or the child), which indicates the value of balancing perspectives in policy development. We have discussed the need to ensure significant child and youth participation in research in other forums [81], while scholars and community leaders have also mentioned the importance of including young people in various settings [34, 82] and the deep reflections and insight young people can provide [30]. Projects like the “#CovidUnder19: Children’s Rights During Coronavirus: Children’s Views and Experiences” [83] have provided an opportunity to open space for, highlight, and listen to the voices of young people. However, there must be consistent national priorities to listen to young people’s perspectives and meaningfully include young people in policy conversations, including but not limited to pandemic scenarios.

As implied throughout the framework, well-being is broader than merely considering the health impacts a person faces; well-being encompasses various aspects of a person’s experiences living in the world. As such, ethical frameworks for use in a pandemic need to attend to the ways in which social determinants of health have crucial importance for the overall well-being of a person and a society. Research on the “Health in All Policies” approach has highlighted the need for an attuned and systematic focus to examine the health and health system impacts of all public policies in an attempt to “improve population health, health equity and the context in which health systems function” [84p. 6]. Health transcends the health sector and the field of medicine, and, therefore, decisions outside the health sector have crucial impacts for overall health and well-being outcomes of a society. Many of the adaptations in the framework highlight other sectors that have a crucial importance for the well-being of young people in a pandemic and how an ethical framework with a child-inclusive lens can account for these factors.

Importantly, although childhood ethics and children’s rights lenses are child-inclusive, our approach within this adaptation was to provide a more collectivist picture where young people are truly acknowledged to be within that collective, not outside of it. This means requiring concessions from young people and child services organizations for the sake of broader public health interests, but simultaneously providing young people with supports for their own interests and rights to be acknowledged and supported. This entails taking steps to ensure that the least restrictive options are used, where the restrictions include those imposed onto both adults *and* children.

### Actualizing this re-envisioned framework in practice

The following two examples have been adapted by the authors to illustrate how our child-inclusive framing of Thompson et al.’s framework can be applied in the real-world. The first example strongly aligns with intended use of the Thompson et al. framework—as a health care resource allocation framework [7].

1. *Access to limited health care resources:*
  Some pandemics raise concerns about the demands for selected crucial limited-supply resources—typically ICU care and mechanical ventilation—which may exceed the available capacities, even when all possible mobilization measures have been maximized. However, there are also questions about the relative priority of children and adults, in light of the relatively lower risk of severe illness that children have faced in the COVID-19 pandemic compared to adults.
  When planning local and regional public health measures, how should children be prioritized in relation to adults in general and more specifically in terms of children with different types of illnesses and/or disabilities?

An ethically-driven response to the case described above, guided by the adapted Thompson et al. framework, indicates that we must recognize the proportionally lower infections risks that a child may face compared to an adult, while ensuring that a child’s rights are treated as equally important as matters of an adult’s autonomy. While there is a definitive need to protect all members of society from various forms of harm (including, but not limited to, viral transmission), the notion of stewardship means that when resources can be shared between populations they should be. This is applicable to the case above, as children have, on the whole (though there are exceptions), done significantly better in relation to hospitalization and mortality rates from COVID-19 compared to adults. As such, an ethical response requires that some resources from pediatric care institutions be shared with adults in general. However, as mentioned, there are exceptions to the cases of children doing better than adults and this is particularly true for children with disabilities and those with underlying health conditions. These young people who are facing higher risks, in proportion to the population of young people as a whole, must be granted greater priority in accessing and preserving resources. As such, it is not simply permissible to utilize pediatric resources to save the adults facing higher transmission risks, as the young people who also face significant transmission risks cannot be overlooked and their pre-existing health needs must also be granted priority to ensure their rights to care are protected and their harms are not disproportionate compared to the rest of the population. Amongst the other principles, this process must be driven by transparency with providers and families and by responsiveness to the variable needs, interests, and risks of populations. Young people, including those with disabilities, should be consulted to understand their concerns in this context.

The second example aligns with the approach we have taken in this paper, wherein the notion that health resource management begins and ends in the realm of medicine is challenged. Instead, investment of social supports, educational, community, and parental resources is emphasized, as each contribute to the health and overall well-being of young people.

2. Lockdown impacts:
  The COVID-19 pandemic has revealed that lockdowns can be highly effective in preventing viral transmission within communities, but can also lead to sometimes severe (sometimes apparently irreparable) social and mental health harms for young people. For example: social impacts have been borne by children/youth, particularly those from low-income households, as a result of school lockdowns and there are heightened challenges in accessing mental health services and community programs for this population.
  When planning local, regional, or national public health measures for pandemic management, how should social and mental health impacts be considered in relation to the biological impacts of viral transmission?

Responding to this case in an ethically sound way driven by the adapted framework requires, first and foremost, being willing to broaden the definition of harm to encompass other forms of risk, as defined in the “protection of the public from harm” principle. By doing this, we acknowledge that transmission-related risks are crucial to contain, but there are several impacts that are more severely affecting many young people (social impacts, education impacts, and others) that need to be minimized by a dedicated subset of resources. A balanced, proportionate, and ethical approach means weighing the entire scope of impacts affecting young people, especially considering the ways in which transmission impacts have often been found and framed to be minimally affecting young people. We must ask: what risks are they facing and how do we weigh these risks despite the different timelines they may be occurring on? Fundamentally, framing these resource investments as an expanded “duty to provide care”, and with a new take on what we mean by stewardship, allows mitigation strategies to be prioritized beyond merely the health care field. Without this, we risk allowing young people to face a disproportionate degree of harm in the immediate- and long-term, reducing reciprocity in the response. And processes for accountability, inclusiveness, transparency and reasonableness are all encompassed when we response to social impacts in a purposeful way. Ultimately, while some may say that these resource investments are impractical because of the finite resources that any country has access to, we encourage stakeholders to consider whether these investments may still be practicable by considering the ways in which resource shifting is a constant in the context of government budgets. Young people’s experiences in the pandemic need to be included, because exposing them to risks beyond the minimal threshold for that risk is a profound concern and a choice that exposes these plans to ethical critique.

### Challenges and limitations

The work to expand this framework did face some challenges and limitations that ought to be accounted for. First, this expanded framework was constructed while still within a pandemic, which is distinctive from the way that Thompson and colleagues completed their work [7]. The benefit of this approach is that we were able to draw on reflections and cases that were recent and still pertinent, leading to a heightened importance for this work. However, the challenge has been that findings on pandemic impacts are still emerging and lessons are still being drawn. As such, we would highlight the need for future work to engage in some retrospective reflection on the lessons and advances discussed in this paper and the utility of this framework.

Second, our work posits a challenge to the status quo or the dominant discourse present in most societies by supporting a view that children deserve a fair footing at policy tables and a chance to meaningfully participate, be heard, and be acknowledged. And yet, we are operating from a critical position while still being situated within the dominant discourse that undermines children’s capacities and deprioritizes their rights; this makes the task of driving change feel overwhelming. However, we recognize that small steps lead to change and this framework is a step in the right direction.

Third, our work fills gaps explicitly mentioned by Thompson et al. [7], namely by engaging youth and other key stakeholders (including some parents) for feedback on the core elements of our proposed framework. In engaging with youth, we consulted one non-specialized youth advisory group as stakeholders to provide broad perspectives related to young people’s experiences. However, we recognize the limitations associated with engaging with a small number of youth. In the future, it would be useful to engage more children and youth as stakeholders in the process of evaluating this framework, but also as research participants who can discuss the ways in which this framework aligns with the care they received during the pandemic and/or the pandemic policies that touched their lives. Ideally, a qualitative inquiry involving a participatory-action methodology would be utilized in future work.

Finally, context and environment can have a massive impact on the ways in which ethical principles are interpreted and a childhood ethics lens is applied. As such, we encourage readers to consider how this framework needs to be adapted based on context. This critical reflection and revision aligns with the epistemological orientation driving this work, of social constructivism and the acknowledgement, as identified earlier in the paper, that there are no universal experiences of childhood.

## Conclusion

In this paper, we have re-envisioned the ethical processes and principles presented by Thompson and colleagues [7] with a “young person focus” that is informed by children’s rights literature and a childhood ethics framework. We have also highlighted that ethical frameworks for pandemic decisions should be inclusive of impacts affecting all aspects of a person’s well-being, rather than solely health sector impacts, for a sound ethical framework. Efforts to ensure frameworks are truly child-inclusive should be the status-quo, meaning the implications can be considered well in advance of emergency preparedness contexts. With this in mind, we agree with a final thought mentioned by Thompson et al., namely that “values are not static” [7]—that pandemic circumstances can rapidly evolve and that different settings may lead to different forms of the framework’s implementation. As such, consistent re-evaluation and revision, as we have undertaken in this work, is necessary to ensure frameworks are context specific and continually valuable. Importantly, it must be noted that a pandemic framework is not a panacea and those deciding how resources are to be allocated will still be required to make difficult choices. Our hope is that this framework provides a starting point to guide these tough decisions—whether in response to ongoing concerns related to the COVID-19 pandemic or for future public health emergencies that put young people’s interests, rights, and lives at risk—and initiate discussion in a young person-inclusive manner.

## Data Availability

Not applicable.

## Abbreviations

CRC: United Nations Convention on the Rights of the Child
CRIA: Children’s Rights Impact Assessment

## Footnotes

1 For more information on the processes and principles/values articulated by Thompson and colleagues, we highly recommend reviewing tables 1 and 2 in their paper (7).

2 The term ‘interests’ is used throughout the paper to align with the notion of ‘best interests’ that is pervasive to writings about pediatric health, well-being, and ethics. To be clear, though, interests refers to the fundamental interests a young person has, their needs, rights, views, and aspirations. As aforementioned, childhood ethics also challenged the ways in which ‘best interests’ are understood and pushes against the adult-centred perspective of this concept.

3 Inclusiveness is often positioned as a key value in framework developments. However, the notion of inclusiveness itself can often fail to sufficiently capture the structural elements that require dismantling and the true recognition of power differentials that exist across a group. As such, we retain this value in this framework but emphasize that our conception of inclusion implies a co-creation process, whereby ‘levelling’ of power is a priority.
